# Small-Molecule Inhibition of CBX4/7 Hypersensitises Homologous Recombination-Impaired Cancer to Radiation by Compromising CtIP-Mediated DNA End Resection

**DOI:** 10.3390/cancers16112155

**Published:** 2024-06-06

**Authors:** Hugh C. Osborne, Benjamin M. Foster, Hazim Al-Hazmi, Stefan Meyer, Igor Larrosa, Christine K. Schmidt

**Affiliations:** 1 Manchester Cancer Research Centre (MCRC), Division of Cancer Sciences, School of Medical Sciences, Faculty of Biology, Medicine and Health (FBMH), University of Manchester, 555 Wilmslow Road, Manchester M20 4GJ, UK; osborne_hugh@hotmail.com (H.C.O.); benjamin.foster@manchester.ac.uk (B.M.F.); hazim.alhazmi@postgrad.manchester.ac.uk (H.A.-H.); stefan.meyer@manchester.ac.uk (S.M.); 2 Department of Paediatric and Adolescent Oncology, Royal Manchester Children’s Hospital, Manchester M13 9WL, UK; 3 Department of Adolescent Oncology, The Christie NHS Foundation Trust, Wilmslow Road, Manchester M20 4BX, UK; 4 Department of Chemistry, University of Manchester, Chemistry Building, Oxford Road, Manchester M13 9PL, UK; igor.larrosa@manchester.ac.uk

**Keywords:** cancer, DNA repair, DNA end resection, homologous recombination (HR), CtIP, UNC3866, CBX4

## Abstract

**Simple Summary:**

DNA damage occurs in healthy cells and cancer cells, but the quantity of damage and how efficiently it is repaired often differ between them. Due to genetic alterations, cancer cells can become ‘addicted’ to one or more forms of repair. This can be exploited by blocking DNA repair pathways that healthy cells can repair via redundant methods which are abrogated in cancer cells. Here, we identify such a scenario using a small molecule targeting CBX4, a protein key to a subset of DNA repair processes important for mending DNA double-strand breaks (DSBs). Consistent with the inhibition of these processes, treatment with the compound results in the selective killing of certain cancer cells when combined with ionizing radiation (IR). These findings raise the possibility that targeting CBX4 and/or the DSB repair pathways it regulates might be exploited more generally for the development of targeted anti-cancer strategies in the future.

**Abstract:**

The therapeutic targeting of DNA repair pathways is an emerging concept in cancer treatment. Compounds that target specific DNA repair processes, such as those mending DNA double-strand breaks (DSBs), are therefore of therapeutic interest. UNC3866 is a small molecule that targets CBX4, a chromobox protein, and a SUMO E3 ligase. As a key modulator of DNA end resection—a prerequisite for DSB repair by homologous recombination (HR)—CBX4 promotes the functions of the DNA resection factor CtIP. Here, we show that treatment with UNC3866 markedly sensitises HR-deficient, NHEJ-hyperactive cancer cells to ionising radiation (IR), while it is non-toxic in selected HR-proficient cells. Consistent with UNC3866 targeting CtIP functions, it inhibits end-resection-dependent DNA repair including HR, alternative end joining (alt-EJ), and single-strand annealing (SSA). These findings raise the possibility that the UNC3866-mediated inhibition of end resection processes we define highlights a distinct vulnerability for the selective killing of HR-ineffective cancers.

## 1. Introduction

The targeting of DNA repair pathways is an emerging concept in cancer therapy as acquired imbalances in the DNA damage response (DDR) with over-dependence on specific repair pathways are common acquired vulnerabilities in cancer. Compounds that target specific DNA repair processes are therefore of therapeutic interest [1,2,3]. The repair of DNA double-strand breaks (DSBs), which can be caused by chemotherapy and radiation, is carefully coordinated across the cell cycle. Two DSB repair pathways are mainly employed depending on the cell cycle phase, DSB structure, and repair factor abundance [4,5]. Non-homologous end joining (NHEJ) is active throughout interphase [6,7] and consists of the limited processing of broken DNA ends followed by their ligation [8]. Due to its availability and favourable kinetics, NHEJ is estimated to repair the majority of DSBs in normal mammalian cells [6]. Homologous recombination (HR), the second main pathway for repairing DSBs, is dependent on the presence of a template DNA sequence, usually belonging to a sister chromatid, that is homologous to the DNA surrounding the break site. As such, HR permits error-free repair mainly in cell cycle phases in which a sister chromatid is present, particularly in the S and G2 phases following DNA replication [4]. HR is promoted by DNA end resection, a process by which DSB ends are processed to form single-stranded DNA overhangs which are pared back in search for homology. This process requires nucleoprotein filament formation of DNA, first with RPA and then with the recombinase RAD51, ultimately permitting the accumulation of downstream HR mediators at DSB ends [4]. DNA end resection also antagonises NHEJ, affecting the choice of DSB repair pathway [9], and promotes alternative forms of repair in addition to HR. For example, limited DNA end resection can stimulate alternative end joining (alt-EJ), also known as microhomology-mediated end joining (MMEJ), while a more extensive resection can promote the use of single-strand annealing (SSA). Importantly, recent work has implicated an increase in alt-EJ use as a compensatory mechanism in cells coping with HR deficiency [10]. Typically, alt-EJ usage in healthy cells is very low (0.5–1%), while a substantial compensatory increase in HR-deficient cancers could generate a targetable cancer phenotype [11]. Indeed, the inhibition of polymerase θ, the principal DNA polymerase in alt-EJ, results in the preferential killing of certain HR-deficient cells [12].

**Figure 1:**
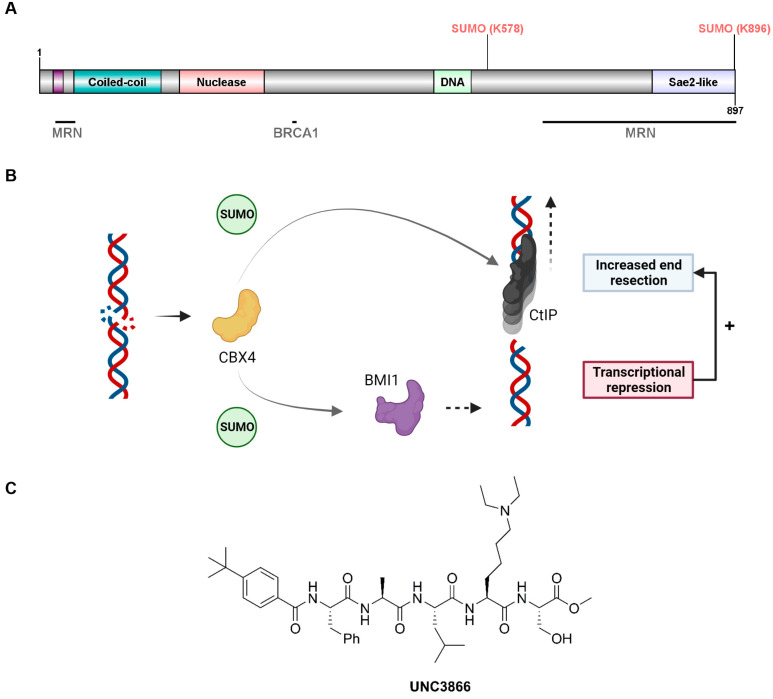
Key functions of CBX4 in CtIP-mediated, end-resection-dependent DNA repair. (**A**) CtIP protein structure, highlighting protein interactors and key post-translational SUMOylation events (K578, K896) relevant to this work. CtIP’s tetramerisation (tet) domain is presented in purple (amino acids 18–31) upstream of the coiled-coil region that mediates CtIP dimerisation. Further regions associated with key CtIP features are displayed in light red (reported endonuclease activity), light green (DNA-binding region), and light blue (Sae2-like domain). (**B**) Graphical model representing distinct steps that CBX4 promotes to fine-tune and optimise DNA end resection. (**C**) Chemical structure of peptidic small-molecule inhibitor UNC3866 targeting CBX4/7. Schematics were generated using BioRender.com (accessed on 24 May 2024).

CtIP (CTBP-interacting protein) is a multivalent adaptor protein in DNA repair that serves as a key end-resection scaffold to promote homology-dependent repair [13,14,15]. It forms a homotetramer that tethers DSB ends to facilitate downstream repair by limiting the conformational freedom of the break site [16] and mediates short-range end resection as an essential co-factor of the central MRN endonuclease complex [15,17]. CtIP promotes both HR and SSA long-range resection mediated by the exonucleases EXO2 and DNA2 [18]. The complete loss of CtIP function leads to a reduction in end-resection-dependent repair efficiency and a compensatory increase in the rate of NHEJ usage [13,14,19,20,21]. CtIP function is governed by post-translational modifications (PTMs), such as distinct phosphorylation events mediated by the apical DDR kinases ATM and ATR, as well as cyclin-dependent kinases (CDKs) (Figure 1A), thereby linking end resection to cell cycle progression [15]. CtIP activity and accumulation at sites of DNA damage are modulated by other PTMs as well, such as ubiquitylation [22], and modification with the small ubiquitin-like modifiers SUMO1 and SUMO2/3 [23,24] (Figure 1A). The SUMOylation of CtIP at the C-terminal lysine K896 promotes CtIP-associated end resection and RAD51 filament formation downstream during HR. This SUMOylation is mediated by the chromodomain protein and the SUMO E3 ligase CBX4 and has been suggested to mark a molecular switch that enables CtIP functioning towards end resection [23]. Proper CBX4 functioning at DSBs depends on its effective recruitment to sites of DSBs, which requires its chromodomain [25]. The SUMOylation of K896 in concert with the PIAS4-mediated SUMOylation of CtIP on lysine K578 represent two well-characterised SUMOylation sites on CtIP [23,24]. In addition to promoting CtIP function via SUMOylation, CBX4 enhances CtIP-mediated resection more indirectly by SUMOylating the upstream DSB response protein BMI1 (Figure 1B). The SUMOylation of BMI1 promotes its recruitment to DSBs to facilitate the BMI1-mediated ubiquitylation of histone H2A on lysine K119 (H2AK119). This in turn then promotes CtIP recruitment to DSB-surrounding chromatin, leading to increased rates of end-resection-dependent DSB repair which are reflected by increased RPA foci formation [26].

While CtIP represents an attractive drug target because of its specific roles linked to end resection processes [15,27], developing direct CtIP inhibitors is challenging due to its disordered structure and lack of conventional binding pockets [27]. However, targeting factors that sustain CtIP functionality at DSBs represents an alternative approach. CBX4, which promotes the DSB-associated functions of CtIP, is therefore an interesting target. While a small-molecule inhibitor of CBX4 named UNC3866 has been established (Figure 1C) [28], its effectiveness in impacting the DDR remains unexplored. This peptidic small molecule targets the chromodomains of CBX4 as well as its closely related homologue CBX7 with equipotent binding (*K*_d_~100 nM), consistent with the partial sequence homology of CBX4 and CBX7, without not exhibiting a specific affinity for other CBX or related proteins [28]. While CBX4 has been linked to the DNA damage response as outlined above, the direct roles of CBX7 in DNA repair have not been uncovered.

Here, we use UNC3866 towards the inhibition of DNA-end-resection-dependent processes. We show that UNC3866 treatment reduces end resection levels and IR-induced RAD51 foci formation in the S and G2 phases, consistent with reductions in both HR and alt-EJ repair. Strikingly, in cancer cells displaying HR defects and upregulated NHEJ, UNC3866 treatment selectively resulted in marked IR sensitisation. By contrast, UNC3866 treatment has little to no effect on cells with intact HR regardless of IR. Our findings have the potential to open up novel avenues of selectively targeting HR-compromised cancers based on the chemical inhibition of CBX4-mediated DDR functions.

## 2. Materials and Methods

### 2.1. Cell Culture

Cell lines were cultured at 37 °C in a humidified 5% CO_2_ atmosphere. U2OS (RRID: CVCL_0042) and U2OS-derived cells (DR-GFP, EJ2-GFP and EJ7-GFP, kindly provided by Jeremy Stark (Beckman Research Institute of the City of Hope, Duarte, CA, USA)) [29] were grown in high-glucose Dulbecco’s Modified Eagle Medium (DMEM; Sigma Aldrich, St. Louis, MO, USA, cat#D6546) supplemented with 10% (*v*/*v*) foetal bovine serum (FBS; Sigma Aldrich, St. Louis, MO, USA, cat#10270106), 100 U mL^−1^ of penicillin (Gibco, Waltham, MA, USA), 100 μg mL^−1^ of streptomycin (Gibco, Waltham, MA, USA), and 2 mM of glutamine (Gibco, Waltham, MA, USA). OVCAR3 and Kuramochi cell lines stably expressing GFP-tagged histone H2B as well as OVMANA cells were kindly provided by Stephen Taylor’s lab (University of Manchester, Manchester, UK) and grown in Roswell Park Memorial Institute (RPMI) 1640 Medium supplemented with glutamine (Gibco, Waltham, MA, USA; cat#21875034), 10% (*v*/*v*) foetal bovine serum (FBS; Sigma Aldrich, St. Louis, MO, USA), 100 U mL^−1^ of penicillin (Gibco, Waltham, MA, USA), 100 μg mL^−1^ of streptomycin (Gibco, Waltham, MA, USA), and an additional 2 mM of glutamine (Gibco, Waltham, MA, USA) [30]. All cell lines were routinely authenticated and tested for mycoplasma prior to use and passaged a minimum of one and a maximum of twelve times prior to use.

### 2.2. Plasmids and Transfection

This study employed the plasmids pEGFP-C1 (cat# 6084-1, Clontech, Mountain View, CA, USA) and pQXIX_2NLS-HA-I*Sce*I, cloned using HA-I*Sce*I as a template [31] and inserted into pQXIX. 7a and 7b sgRNA for the EJ7-GFP reporter assay were a gift from Jeremy Stark (Addgene plasmid #113624; RRID: Addgene_113624; Addgene, Watertown, MA, USA). Culture dishes (60 mm) were transfected at ~70% cell confluence. For the DR-GFP U2OS cells, Fugene6 (13.2 μL, cat#E2691; Promega, Madison, WI, USA) was added to warmed OptiMEM (300 μL; Gibco, Waltham, MA, USA) and mixed gently; the mixture was then incubated at room temperature (RT) for 5 min, followed by addition of the plasmid (4 μg) and gentle mixing. The mixture was incubated at RT (20 min). Following the aforementioned incubation, the mixture was added drop-wise to the dish. To minimise transfection toxicity, the cell growth medium was replaced after 24 h. For the EJ2-GFP and EJ7-GFP U2OS cells [29], a plasmid (4 μg) was added to warmed Opti-MEM (250 μL; Gibco, Waltham, MA, USA) and mixed gently. Then, Lipofectamine 2000 (10 μL, cat# 11668019; Invitrogen, Waltham, MA, USA) was mixed gently into the above mixture (RT; 5 min). Then, the lipid mixture was gently combined with the DNA mixture and incubated (RT; 20 min). Prior to transfection, the cell growth medium was replaced with fresh growth medium without antibiotic supplementation (2 mL). Following incubation, the nucleic acid–lipid mixture was added drop-wise to the dish. To minimise transfection toxicity, the cell growth medium was replaced after 16–24 h.

### 2.3. Compounds Used in Biological Assays

Stock solutions for 5-ethynyl-2′-deoxyuridine (EdU; cat#C10339; Invitrogen, Waltham, MA, USA; 10 mM), 4′,6′-diamidino-2-phenylindole dihydrochloride (DAPI; Acros organics, Geel, Belgium; 1 mg mL^−1^), the CBX4/7 inhibitor UNC3866 (cat#19237-5 mg-CAY; Cayman Chemicals, Ann Arbor, MI, USA; 20 mM), the ATM inhibitor (ATMi) KU55933 (cat#A4605-APE-10 mg; Stratech Scientific, Ely, UK; 10 mM), the SUMO E1 inhibitor (SUMOi) ML-792 (cat#407886; Medkoo Biosciences, Durham, NC, USA; 1 mM), the PARPi olaparib (cat#A10111-10; Generon, Houston, TX, USA; 100 mM), ATRi AZD6738 (cat#B6007; APExBIO, Houston, TX, USA; 10 mM), camptothecin (CPT; Sigma Aldrich, St. Louis, MO, USA; 10 mM), DNA-PKi AZD7648 (#S8843-SEL-5 mg; Stratech Scientific, Ely, UK; 10 mM), and WEE1i AZD1775 (cat#21266; Cayman Chemicals, Ann Arbor, MI, USA; 10 mM) were prepared in dimethyl sulfoxide (DMSO). A stock solution of hydroxyurea (HU; cat# H8627; Sigma Aldrich, St. Louis, MO, USA; 1 M) was prepared in double-distilled water.

### 2.4. Plasmid Re-Joining Assays

For HR activity, DR-GFP U2OS cells, a U2OS-derived cell line stably expressing an inactive GFP cassette containing a cleavage site recognised by the I-*Sce*I endonuclease (SceGFP), were used [32]. The cleavage site contains a stop codon so that functional GFP is not expressed from the above cassette. Downstream of this site lies a truncated GFP coding sequence (tGFP) spanning the section that is missing in SceGFP. The transfection of this stable cell line with a plasmid expressing the I-*Sce*I endonuclease results in the cleavage of the SceGFP cassette, generating a DSB. Should this DSB be repaired using HR with the tGFP cassette acting as a template, the GFP open reading frame is restored. GFP expression in cells was then quantified using flow cytometry [33,34]. To measure alt-EJ repair events, we used U2OS cells stably expressing the EJ2-GFP reporter [19]. In this system, the I*Sce*I-targeted restriction site and an ensuing stop codon are flanked by 8-nucleotide stretches of microhomology. The major alt-EJ product (~85%) following I*Sce*I-mediated cleavage results from the recognition of these microhomologies, leading to a 35-nucleotide deletion that encompasses both the above restriction site and stop codon and restores a GFP open reading frame. Two minor end-joining products with restored GFP expression also occur following restriction site cleavage and repair: one is generated from a 23-nucleotide deletion with no microhomologies (~15%), and the other involves much more extensive deletions (140–350 nucleotides) but only represents a small minority of repair events (~5%) [19,33]. To measure distal NHEJ repair events, we used U2OS cells stably expressing the EJ7-GFP reporter [35]. The reporter has a 46 bp insertion within the GFP coding sequence which is removed upon transfection with two sgRNAs (7a and 7b), generating a blunt-ended DSB. The restoration of the GFP-coding sequence and subsequent GFP fluorescence, as measured and quantified using flow cytometry, are achieved through c-NHEJ indel-free repair.

### 2.5. Flow Cytometry

For a DNA content analysis, the cells were treated as indicated and then harvested according to experimental conditions. The cells were washed in PBS and then counted. All samples were resuspended in a small volume of PBS and gently vortexed while 70% ethanol was slowly added. The cells were fixed overnight (4 °C). Following this, the samples were washed in PBS and then resuspended in 0.5% Triton X-100 in PBS, RNase A (200 μg mL^−1^; Sigma Aldrich, St. Louis, MO, USA), and propidium iodide (20 μg mL^−1^; Sigma Aldrich, St. Louis, MO, USA), before incubation (2 h; 4 °C). Finally, the cells were resuspended in PBS and stored (4 °C) prior to analysis or analysed immediately using an Invitrogen Attune NxT flow cytometer (Thermo Fisher Scientific, Waltham, MA, USA). A total of 10,000 cells were analysed per condition using FlowJo (LLC, Ashland, OR, USA; RRID: SCR_008520). For plasmid re-joining assays, cells (DR-GFP U2OS, EJ2-GFP U2OS or EJ7-GFP) were pre-treated as indicated. Following DMSO or UNC3866 pre-treatment, the cells were transfected with mock (omitting plasmid), eGFP-C1 (4 μg), pQXIX_2NLS-HA-ISceI (4 μg), or sgRNA 7a/7b (4 μg total DNA), according to the Fugene6 (DR-GFP U2OS) or Lipofectamine 2000 (EJ2-GFP or EJ7-GFP U2OS) protocols outlined above (Section 2.2). For pulse drug treatments (KU55933 in DR-GFP U2OS; olaparib in EJ2-GFP U2OS), the corresponding DMSO stock volume was added drop-wise to the existing dish volume 2 h after transfection. After 16–24 h, the cells were rescued with the addition of fresh medium (4 mL). After a further 24 h, the cells were harvested using trypsin–EDTA (0.05%; cat#25300-054; Gibco, Waltham, MA, USA). This was neutralised in the appropriate cell growth medium prior to centrifugation (200× *g*; 5 min) and supernatant aspiration. The cells were washed in PBS (1 × 10 mL). Live cells were then re-suspended in PBS (0.5 mL) and analysed using an Invitrogen Attune NxT flow cytometer (Thermo Fisher Scientific). A total of 10,000 cells were recorded and analysed per condition using FlowJo (FlowJo, LLC). The final GFP-positive population per condition was determined by correcting for transfection efficiency and normalised to the DMSO-treated cells.

### 2.6. Live-Cell Imaging

Cells were seeded in 96-well plates (Greiner Bio-One, cat#655087, Frickenhausen, Germany, or Perkin-Elmer, cat#6005550, Waltham, MA, USA) 24 h before drug treatment. The following day, the cells were treated with the requisite concentration of drug and further incubated (24 h for UNC3866; 2 h for others and for drug combination experiments), at which point designated plates were irradiated (2 Gγ) using a Faxitron CellRad irradiator (Faxitron Bioptics, LLC, Tucson, AZ, USA). The cells were imaged using an IncuCyte S3 system (Essen BioScience, Ann Arbor, MI, USA; RRID: SCR_019874) at 20× magnification in a humidified 5% CO_2_ atmosphere at 37 °C every 4 h. At each time-point for individual conditions in each well, 9 images were acquired. The green fluorescent object count (GOC) of each of the GFP-H2B modified cell lines was quantified via a real-time, semi-automated analysis. Data were exported from IncuCyte software (v2020C or 2022B Rev2), processed in Microsoft Excel (version 365), and graphed using Prism 8 (GraphPad, San Diego, CA, USA; RRID: SCR_002798). Proliferation curves show the mean ± standard error of the mean (SEM) values of the cell counts from 9 images per well in triplicate wells (*n* = 27).

### 2.7. High-Content Screening and Immunofluorescence

Cells were seeded into 96-well plates (Perkin Elmer; cat#6005550) for 24 h; after this period, they were treated with the requisite concentration of drug for the indicated period of time. Following this, designated plates were irradiated using a Faxitron CellRad irradiator (Faxitron Bioptics, LLC, Tucson, AZ, USA); for irradiation conditions, see the corresponding Figure legends. For non-pre-extracted samples (anti-RPA1 and anti-RAD51), the growth medium was removed and the cells were fixed in 2% paraformaldehyde in phosphate-buffered saline (PBS; RT, 20 min), permeabilised using 0.5% Triton X-100 in PBS (15 min; RT), and then stored in 0.1% Tween-20 (Promega) in PBS (hereafter referred to as PBST) at 4 °C. PBST washes (2–3×) were performed before and after each step. The cells were then stained using primary antibodies and the corresponding anti-rabbit or anti-mouse secondary antibody combined with DAPI. After each antibody incubation, the cells were washed in PBST (2–3×).

#### 2.7.1. Image Acquisition and Analysis

Images were acquired employing an Operetta high-content imaging system (Perkin-Elmer) with a 63× water objective and quantified using Columbus high-content imaging and analysis software v2.5 (Perkin-Elmer). Nuclear foci numbers were quantified as 96-well plate well averages and graphed using Prism 8 (GraphPad). Fluorescence images were prepared using ImageJ v1.52a (NIH, Stapleton, NY, USA).

#### 2.7.2. Manual Counting

Where semi-automated analysis was not possible (RAD51 foci in EdU^+^ cells), manual counting of the above features was undertaken. For RAD51 foci in EdU^+^ cells, 50 EdU^+^ cells per technical replicate were analysed (i.e., in triplicate—150 EdU^+^ cells per biological replicate). Data are presented as a single mean value for each biological replicate.

### 2.8. Antibodies for Immunofluorescence

The antibodies used were anti-RAD51 (Bio-Academia, Osaka, Japan; cat#70-001; 1:6000), anti-RPA1 (Abcam, Cambridge, UK; cat#ab79398 and RRID: AB_1603759; 1:500), anti-rabbit Alexa Fluor 488 IgG (H + L) (Invitrogen, Waltham, MA, USA; cat#A27034 and RRID: AB_2536097; 1:500), anti-mouse Alexa Fluor 488- and anti-mouse Alexa Fluor 594-conjugated IgG (H + L) (Invitrogen, Waltham, MA, USA; cat#A28175 and RRID: AB_2536161; cat#A11032 and RRID: AB_2534091, both 1:500). They were diluted in PBST and used as described above.

### 2.9. Statistical Analysis

Statistical analyses were performed using Prism 8 (GraphPad, San Diego, CA, USA; RRID: SCR_002798). Unless otherwise stated, data were used as generated or mean (±SEM) values were calculated based on independent experiments. Statistical significance between two groups or for normalised repair efficiency data was determined using the non-parametric two-tailed Welch’s *t*-test, and statistical significance between three or more groups was determined using a parametric one-way ANOVA followed by Tukey’s multiple-comparisons test when *n* = 3. When *n* ≥ 30, statistical significance between three or more groups was determined using a non-parametric one-way ANOVA for unequal variances followed by the Games–Howell multiple-comparisons test in accordance with the central-limit theorem and ANOVA robustness studies [36]. For all multiple comparisons, the multiplicity adjusted *p*-value was reported as non-significant (ns)—*p* > 0.05, *—*p* < 0.05, **—*p* < 0.01, ***—*p* < 0.001, and ****—*p* < 0.0001.

## 3. Results

### 3.1. UNC3866 Decreases DNA End Resection Efficiency

**Figure 2:**
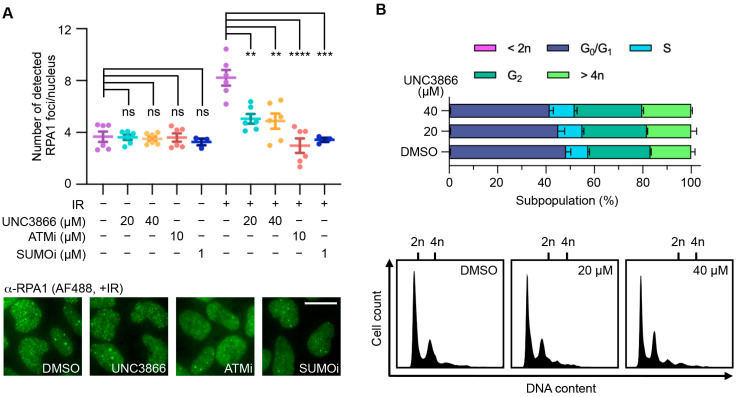
UNC3866 reduces DNA end resection efficiency in U2OS cells. (**A**) U2OS cells were treated with UNC3866, KU55933 (ATMi), ML-792 (SUMOi), or the vehicle only (DMSO) at the indicated concentrations 72 h before being irradiated (10 Gγ; 1 h recovery) or not. The cells were subsequently stained for RPA1 foci and their numbers analysed as indicated (Alexa Fluor 488—AF488). The top graph shows data collected over *n* = 3–6 replicates, each representing the result from one 96-plate well average. Means are highlighted as horizontal lines. Representative immunofluorescence images are shown at the bottom. The scale bar indicates 10 μm. (**B**) A cell cycle analysis of propidium iodide-treated U2OS cells after 72 h of treatment with UNC3866. Histograms (**top**) and representative cell cycle distributions depicting the DNA content (**bottom**) are shown (*n* = 3; means ± SEMs). Statistical significance, or not, is indicated as follows: ns: non-significant (*p* > 0.05); **: *p* < 0.01; ***: *p* < 0.001; ****: *p* < 0.0001.

To evaluate whether the UNC3866 treatment affected CBX4 activity in relation to its functional links to both CtIP and BMI1 in DNA end resection, we analysed RPA foci formation in cells, which is heavily affected by the disruption of CtIP function [37]. The UNC3866 treatment of U2OS cells induced a significant reduction in RPA1 foci following IR-induced DNA damage (Figure 2A), implying a reduction in end resection activity. In this context, it is noteworthy that the relatively high concentrations of UNC3866 (up to 40 μM) used are representative of the fact that intracellular concentrations of UNC3866 correspond to only approximately 5% of its extracellular concentrations [28]. Moreover, we note that the ionising radiation we employed was used as a means of inducing DNA damage rather than to highlight a prospective treatment modality. The ATM inhibitor KU55933 and the SUMO E1 inhibitor ML-792 (SUMOi), which were previously shown to affect end resection as measured by RPA foci formation [23,24,26], were used as positive controls (Figure 2A). As DSB end resection is highly cell-cycle-dependent, we studied the effects of UNC3866 on cell cycle distribution. UNC3866 caused only minor changes in cell cycle distribution, showing that UNC3866’s effects on end resection were not merely due to alterations in cell cycle progression (Figure 2B).

### 3.2. UNC3866 Reduces End-Resection-Dependent Repair Efficiency

**Figure 3:**
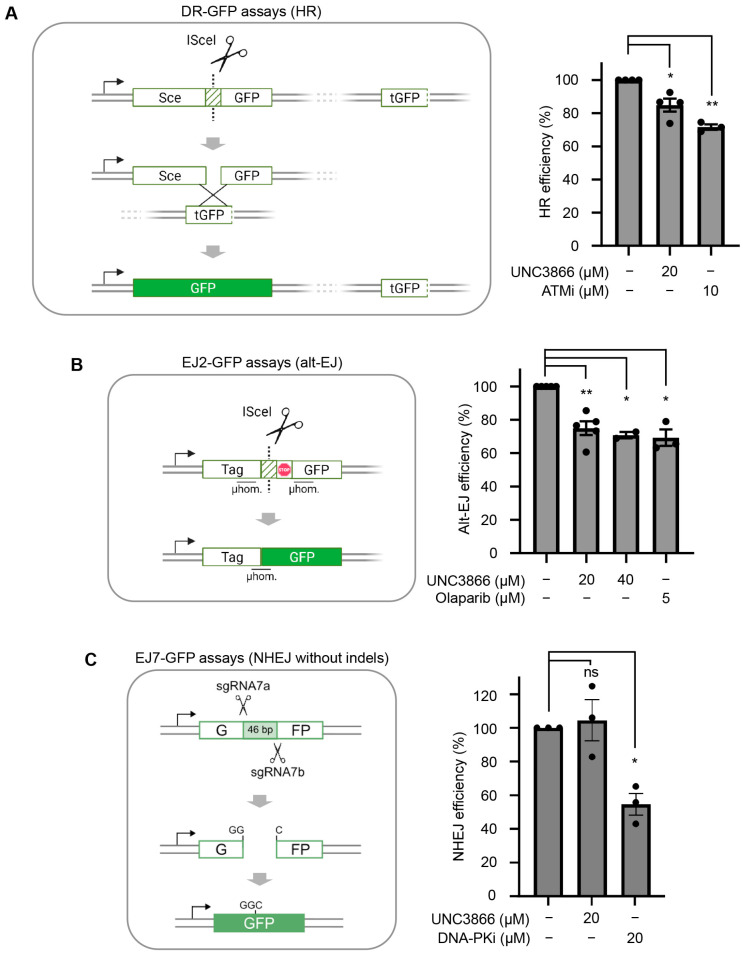
UNC3866 antagonises DNA end-resection-dependent repair. (**A**) Left: A schematic of the fluorescent DR-GFP reporter system for measuring homologous recombination (HR) efficiency in cells using flow cytometry. The I*Sce*I-mediated scission of an interrupted GFP coding sequence (SceGFP) results in the restoration of the GFP cassette if repaired via HR using a downstream truncated GFP sequence (tGFP) as a template. Right: the quantification of GFP-positive cells (%) as detected via a flow cytometry analysis of U2OS cells stably expressing the DR-GFP cassette following treatment with UNC3866 (20 μM; *n* = 4), KU55933 (ATMi; 10 μM; *n* = 3), or the vehicle only (DMSO; *n* = 4). Normalised HR efficiencies are shown as means ± SEMs. (**B**) Left: A schematic of the fluorescent EJ2-GFP reporter system for measuring alt-EJ efficiency in cells using flow cytometry. I*Sce*I-mediated scission of an interrupted GFP-coding sequence results in the restoration of the GFP cassette if repaired via alt-EJ using upstream sequence microhomologies (μhom.) either side of a stop codon. Right: The quantification of GFP-positive cells (%) as detected by a flow cytometry analysis of U2OS cells stably expressing the EJ2-GFP reporter cassette following treatment with UNC3866 (20 μM; *n* = 5, or 40 μM; *n* = 2), olaparib (5 μM; *n* = 3), or the vehicle only (DMSO; *n* = 5). Normalised alt-EJ efficiencies are displayed as means ± SEMs. (**C**) Left: A schematic of the fluorescent EJ7-GFP reporter system for measuring indel-free non-homologous end joining (NHEJ) efficiency in cells using flow cytometry. The CRISPR-sgRNA7a/7b-mediated scission of a 46 bp sequence that interrupts the GFP-coding sequence results in a blunt-ended DSB. Restoration of the GFP cassette is achieved through repair via indel-free NHEJ. Right: A quantification of GFP-positive cells (%), as detected by a flow cytometry analysis of U2OS cells stably integrating the EJ7-GFP reporter cassette following treatment with UNC3866 (CBX4/7i, 20 μM, *n* = 3), DNA-PKi (AZD7648, 20 μM, *n* = 3), or the vehicle only (DMSO, *n* = 3). Normalised NHEJ efficiencies are displayed as means ± SEMs. Statistical significance, or not, is indicated as follows: ns: non-significant (*p* > 0.05); *: *p* < 0.05; **: *p* < 0.01. The schematics were generated using BioRender.com (accessed on 24 May 2024).

To expand on the functional impact of UNC3866 on DNA repair pathways promoted by its DDR target CBX4, we studied the effects of UNC3866 using a set of DNA repair reporters, including DR-GFP to measure HR efficiency (Figure 3A, left) and EJ2-GFP to measure alt-EJ efficiency (Figure 3B, left) [19,32,38].

Employing U2OS cells stably expressing the DR-GFP reporter—an inactive GFP cassette containing a cleavage site recognised by the I-*Sce*I endonuclease (SceGFP)—we quantified the efficiency of DNA repair by HR based on GFP expression, as measured by flow cytometry [32,33,34]. The findings showed that HR efficiency was significantly decreased following the UNC3866 treatment of the DR-GFP U2OS cells (20 μM; 15% reduction) (Figure 3A, right). Given that approximately a quarter of DSBs are repaired by HR across the cell cycle, this reduction likely represents a minor proportion of DSBs (<5%) [6]. The UNC3866 treatment of U2OS cells stably integrating the EJ2-GFP reporter cassette (Figure 3B, left) [19] resulted in a ~30% decrease in alt-EJ repair efficiency (20 μM—25%, 40 μM—29%; Figure 3B, right). This represents a considerable proportion of the reduction in repair efficiency observed by CtIP depletion [19,33]. The impact UNC3866 had on repair efficiency was comparable to the effects of olaparib on alt-EJ (Figure 3B, right), as previously reported, and was consistent with the similar upstream roles of CtIP and PARP1 in alt-EJ, cooperatively facilitating DNA polymerase θ function [19,33]. To test if the efficiency of NHEJ, as another major DSB repair pathway that is independent of CtIP, was affected by UNC3866, we used U2OS cells stably integrating the EJ7-GFP cassette [35]. In this assay, the scission of a 46 bp sequence interrupting the GFP-coding sequence led to a blunt-ended DSB that could be restored through repair mediated by indel-free NHEJ (Figure 3C, left). Treatment with UNC3866 had no marked impact on NHEJ efficiency. The DNA-PKcs inhibitor AZD7648 served as a positive control (Figure 3C, right). Taken together, these results are in line with UNC3866 primarily targeting end-resection-dependent repair pathways that depend on CtIP function.

### 3.3. UNC3866 Selectively Sensitises HR-Deficient Cells to IR

**Figure 4:**
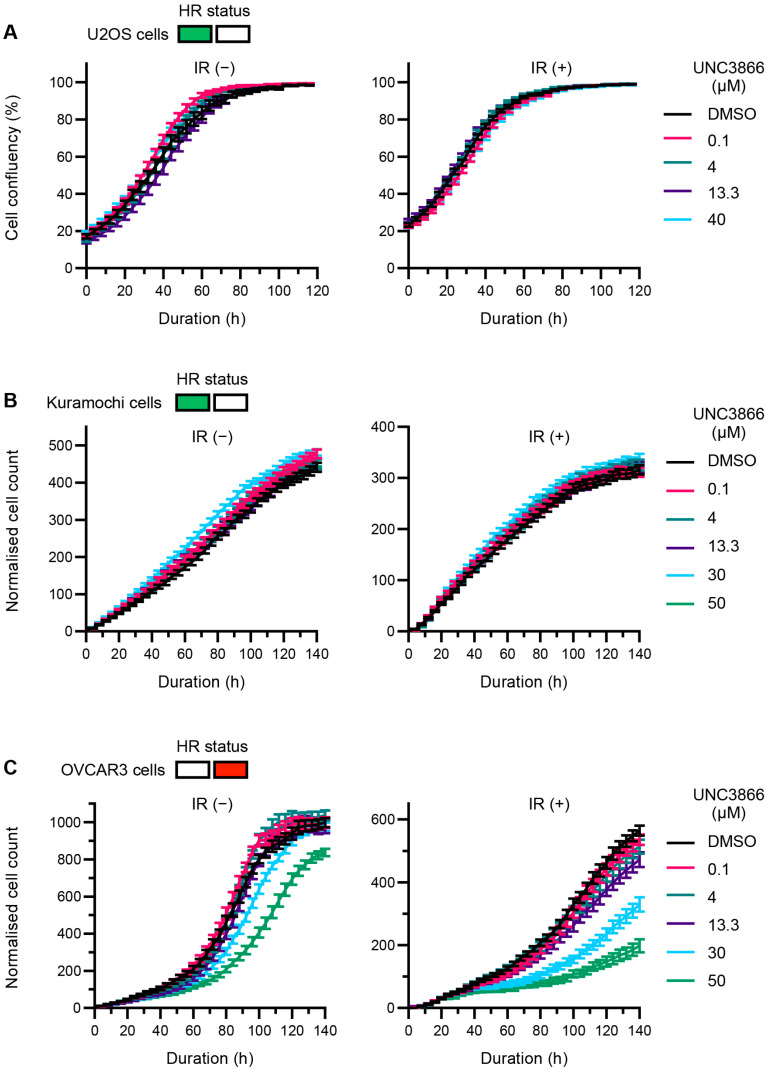
UNC3866 leads to selective cytotoxicity in OVCAR3 cells. (**A**) U2OS, (**B**) Kuramochi, and (**C**) OVCAR3 cells pre-treated with indicated concentrations of UNC3866 or the vehicle only (DMSO; 24 h) were subjected to ionising irradiation (IR; 2 Gγ) or not as indicated, and their growth was tracked for the indicated durations. Representative proliferation curves are shown as means ± SEMs (U2OS—1 independent experiment, Kuramochi—2 independent experiments, and OVCAR3—3 independent experiments). The functional homologous recombination (HR) status is displayed in green for proficient and in or red for deficient.

To further interrogate the effects of UNC3866 on DNA repair, a panel of cell lines was treated with UNC3866 in the presence or absence of IR (2 Gγ). In HR-proficient U2OS (Figure 4A) and Kuramochi (Figure 4B) cells, UNC3866 had no effect even at high concentrations of up to 40 μM, irrespective of additional treatment with IR. In OVCAR3 (high-grade serous ovarian cancer (HGSOC)) cells, however, which exhibit features of HR deficiency and upregulated NHEJ, as described previously [39,40], we observed not only sensitivity to UNC3866 in the absence of IR at higher doses, in particular 50 μM, but also a marked hypersensitisation to IR (Figure 4C). Hypersensitisation to UNC3866 in another ovarian cancer cell line, OVMANA, which also features characteristics of HR deficiency including a *BRCA* mutation and PARPi sensitivity [41], was also observed (Supplementary Figure S1).

### 3.4. UNC3866 Sensitivity Might Derive from Alt-EJ Inhibition Rather Than Replication Fork Destabilisation

**Figure 5:**
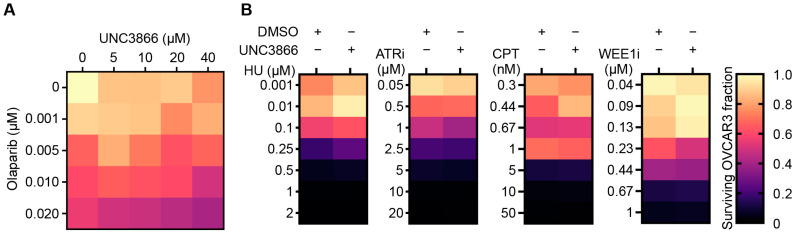
UNC3866 does not synergise with DNA-replication-stress-inducing drugs. (**A**) A heat map representing the proliferation of OVCAR3 cells in the presence of varying concentrations of the PARP inhibitor olaparib and UNC3866 (a 5 × 5 concentration grid, as indicated), normalised to the vehicle only (DMSO; 108 h; *n* = 2). (**B**) Heat maps representing the proliferation of OVCAR3 in the presence of hydroxyurea (HU—*n* = 2), AZD6738 (ATRi—*n* = 3), camptothecin (CPT—*n* = 2), or AZD1775 (WEE1i—*n* = 2) over a range of concentrations with and without UNC3866 (20 μM), normalised to the vehicle only (DMSO; 140 h).

As the disruption of PARP1 activity biases DSB repair towards the use of NHEJ, which is already upregulated in OVCAR3 cells relative to other HGSOC cell lines [39,42,43], we combined UNC3866 with the PARP inhibitor olaparib in a 5 × 5 combinatorial grid to assess their combined effects in HR-defective OVCAR3 cells. Consistent with partial epistasis in alt-EJ, most combinations of the two drugs were not additive and resulted in a surviving fraction equivalent to the lowest induced by either drug alone at any given concentration. Some cooperativity was observed when the highest dose of olaparib was combined with UNC3866 (Figure 5A), perhaps due to off-target or other effects, such as PARP trapping or an increasing lack of break-associated BMI1-mediated transcriptional repression [26,44]. However, it is noteworthy that CtIP was recently demonstrated to have a replication-fork-protecting role to prevent the DNA2-mediated over-resection of regressed fork arms following fork reversal [45] linked to the PIAS4-mediated SUMOylation of CtIP on K578 [24]. We therefore investigated whether UNC3866 synergised with inducers of replication stress, such as hydroxyurea (HU), AZD6738 (ATRi), or camptothecin (CPT). These compounds were used in a range of concentrations in the presence or absence of a single concentration of UNC3866. Moreover, given that WEE1 also prevents the DNA2 over-resection of regressed fork arms by restraining CDK2 activity, we combined AZD1775 (WEE1i) with UNC3866 to investigate if this would affect regressed arm over-resection [46]. No significant synergy was observed, and indeed an increase in the surviving fraction was often apparent following the addition of UNC3866 across the drugs explored (Figure 5B). These findings indicate that UNC3866 is unlikely to markedly affect replication fork stability via CtIP or other means.

### 3.5. UNC3866 Reduces RAD51 Foci Formation in OVCAR3 Cells

**Figure 6:**
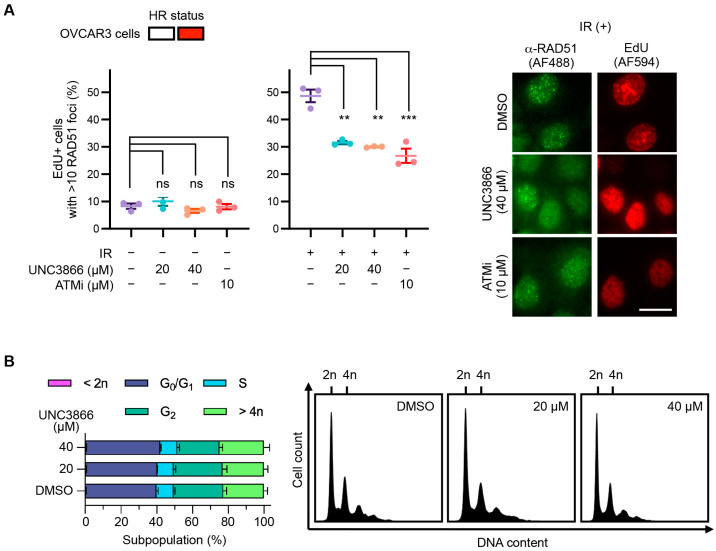
UNC3866 reduces RAD51 foci formation in OVCAR3 cells. (**A**) OVCAR3 cells were treated with UNC3866, KU55933 (ATMi), or the vehicle only (DMSO) at the indicated concentrations for 72 h before treatment with ionising radiation (IR; 4 Gγ and 4 h recovery—(**right**)) or not (**left**). The cells were then fixed and stained for an immunofluorescence analysis of RAD51 foci formation (Alexa Fluor 488—AF488) in S-/G2-phase cells using 5-ethynyl-2′-deoxyuridine (EdU; Alexa Fluor 594—AF594) as a marker. Data represent means ± SEMs (*n* = 3). The scale bar indicates 10 μm. (**B**) A propidium iodide-treated OVCAR3 cell cycle analysis after 72 h of treatment with UNC3866. Histograms (**left**) and representative cell cycle distributions depicting the DNA content (**right**) are shown. Data represent means ± SEMs (*n* = 3). Statistical significance, or not, is indicated as follows: ns: non-significant (*p* > 0.05); **: *p* < 0.01; ***: *p* < 0.001.

While RAD51 foci formation normally provides a functional readout for downstream HR, in OVCAR3 cells, despite exhibiting a functional HR defect, RAD51 foci are formed [39]. In these cells, RAD51 foci formation might indicate that DSBs are being rescued by alt-EJ. Due to the cell-cycle-dependent nature of end-resection-dependent repair, the RAD51 foci analysis was confined to post-replicative S- and G2-phase cells using 5-ethynyl-2′-deoxyuridine (EdU) incorporation and detection. RAD51 immunofluorescence following IR revealed a reduction in foci formation in the UNC3866-treated cells compared to the untreated cells (Figure 6A). At the highest UNC3886 dose used (40 μM), the relative reduction was similar to that elicited by the positive control, the inhibition of ATM (45%). Like in the U2OS cells, the cell cycle distribution was not markedly affected by UNC3866 treatment, confirming that the reduction in RAD51 foci formation was not merely due to modified cell cycle progression (Figure 6B).

## 4. Discussion

There is a paucity of actionable small-molecule inhibitors of DSB repair which could be used as DNA-repair-pathway-targeting therapeutics, such as PARP inhibitors. Therefore, targeting end resection indirectly via CBX4 inhibition using the small-molecule inhibitor UNC3866 [28] is an intriguing prospect. In this work, we studied the effects of UNC3866 in novel DNA repair contexts, highlighting the impact of its CBX4 antagonism with respect to the modulation of CtIP functions. CBX4 is a key constituent of the modular PRC1 complex, as is BMI1, and exhibits SUMO E3 ligase activity [28,47]. CtIP was recently identified as a key target of CBX4, firstly via the SUMOylation of CtIP and secondly through the SUMOylation and consequential stimulation of BMI1, which promotes CtIP end resection activity [23,25,26]. Together, this places CBX4 at the heart of DSB repair pathway choice which, as a critical fine tuner of end resection, forms a PRC1-CtIP-associated repair axis. Given the above, the loss of CBX4 decreases end-resection-dependent repair proficiency [23]. In line with these findings, treatment with UNC3866 to test for its effects on DNA end resection in DNA-repair-proficient cells, revealed reduced rates of resection by RPA foci formation. Functional DNA repair assays further demonstrated that UNC3866 significantly reduced end-resection-dependent repair efficiency with respect to both HR and alt-EJ and coherent with the decrease in RPA foci we observed [33,34]. Given that reliance on these two DSB repair pathways in HR-proficient cells is relatively low, UNC3866 was largely non-toxic in the HR-competent cells we tested. However, cells displaying a well-established HR defect downstream of RAD51 foci formation as well as cells mutated in *BRCA* combined with PARP inhibitor sensitivity were markedly hypersensitised to IR by UNC3866.

Multi-concentration drug–drug combinations of UNC3866 with olaparib in HR-deficient cells resulted in non-additive decreases in cell survival, consistent with a largely epistatic relationship between enzymatic PARP activity and UNC3866-targeted CtIP functions in DSB repair by certain alt-EJ pathways. Interestingly, in plasmid re-joining assays, olaparib treatment or CtIP protein depletion has been shown to abrogate less than half of the measured alt-EJ activity, suggesting that multiple alt-EJ pathways which have not been fully identified as of yet exist, with varying dependencies on CtIP and PARP1 [19,33]. This may explain why higher doses of either drug produced sub-additive cooperativity rather than the total absence of cooperativity often seen at lower dose combinations. Alternatively, higher drug doses may bring other effects to the fore, such as PARP trapping for olaparib or a progressive inhibition of BMI1-dependent transcriptional silencing for UNC3866 [26,44].

As CtIP prevents the DNA2-mediated over-resection of regressed arms at reversed forks, UNC3866 was combined with various inducers of replication stress (HU, CPT, and ATRi) to see if the compound affected CtIP function at replication forks [45,48]. However, UNC3866 did not synergise with any of the above compounds. Taken together, these findings indicate that UNC3866 mainly disrupts end-resection-dependent repair components mediated by CBX4 [23,25,26].

UNC3866 features equipotent binding to both CBX4 and CBX7 [28]. As a PRC1 component itself, CBX7 may cooperate with BMI1 as part of the PRC1 complex, with this activity likely being epistatic with the concurrent inhibition of CBX4-mediated BMI1 SUMOylation which is necessary for BMI1 recruitment to DNA damage [25]. Nonetheless, end resection antagonism may also partially result from the inhibition of non-BMI1-dependent transcriptional silencing, facilitated by CBX7 and redundant ubiquitin E3 ligases that make up the PRC1 complex [49]. Future research is required to further distinguish between these and other possibilities.

## 5. Conclusions

In summary, our data indicate that CBX4 inhibition may be a promising avenue for the development of end-resection-dependent repair antagonists. UNC3866 is strikingly non-toxic in the HR-proficient cell lines studied and, as such, could serve to complement existing treatment modalities for HR-deficient cancers such as PARP inhibitors. Given the distinct target of UNC3866, which is placed upstream of multiple DNA repair pathways, the targeting of CtIP-mediated resection events may feature distinct sensitivity profiles in comparison to PARP and other DDR inhibitors. Future research, including pre-clinical in vivo experiments, is required to further dissect the types of HR-deficient cancers likely to be the most suitable for targeting CtIP activities via CBX4 and how this might synergise with other DDR inhibitors, chemotherapeutics, and/or radiotherapy.

In combination with the structure–activity relationship studies that led to the development of UNC3866 [28], our findings serve as an attractive platform for designing CBX4-selective compounds with optimised pharmacokinetic properties in the future. Such compounds may have potential to contribute towards personalised cancer therapies via the selective targeting of end-resection-dependent DNA repair pathways to sensitise certain HR-defective cancers.

## Data Availability

The data presented in this study are available upon request.

## Supplementary Materials

The following supporting information can be downloaded at: https://www.mdpi.com/article/10.3390/cancers16112155/s1, Figure S1: UNC3866 hypersensitises HR-deficient OVMANA ovarian cancer cells.
