# Supplementary material for: Small-Molecule Inhibition of CBX4/7 Hypersensitises Homologous Recombination-Impaired Cancer to Radiation by Compromising CtIP-Mediated DNA End Resection
Source: Cancers (Basel). 2024 Jun 6;16(11):2155. doi: 10.3390/cancers16112155 (PMC11172190; doi:10.3390/cancers16112155)
Supplement: Supplementary file 1 [file cancers-16-02155-s001.zip › cancers-3000750-supplementary.pdf]

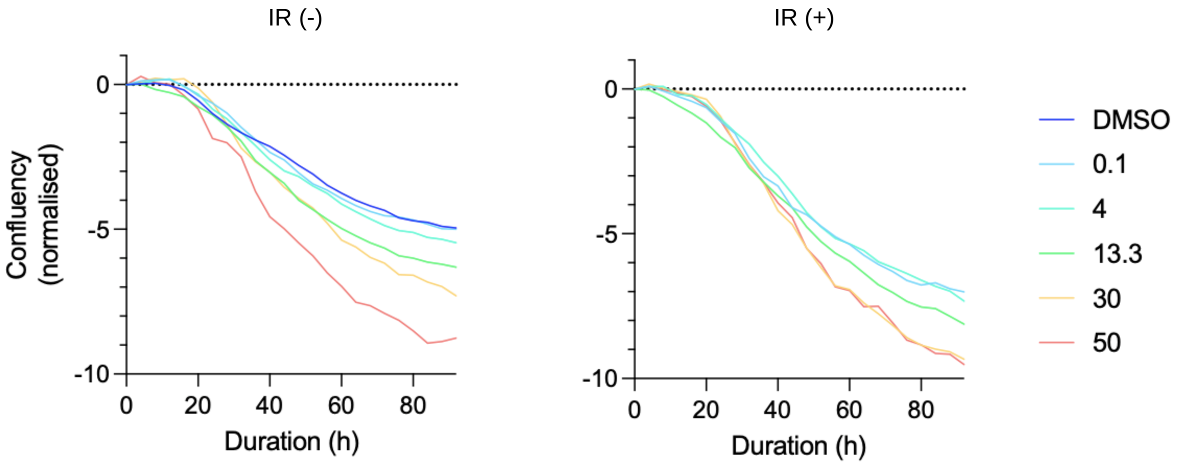

Supplementary Figure S1. UNC3866 hypersensitises HR-deficient OVMANA ovarian cancer cells. OVMANA cells, seeded at low density to assess clonogenic survival potential, pre-treated with the indicated concentrations of UNC3866, or vehicle only, for 24 h, were subjected to ionising irradiation (IR; 2 Gγ) or not as indicated, and their growth tracked for the indicated durations. Normalised confluency curves (means of 3 replicates) are shown.
